# Influence of Fungal Odor on Grooming Behavior of the Termite, *Coptotermes formosanus*

**DOI:** 10.1673/031.010.14101

**Published:** 2010-08-23

**Authors:** Aya Yanagawa, Fumio Yokohari, Susumu Shimizu

**Affiliations:** ^1^ institute of Biological Control, Graduate School of Bioenvironmental Science, Kyushu University, Fukuoka 812- 8581, Japan; ^2^ Research Institute for Sustainable Humanosphere, Kyoto University, Uji 611-0011, Japan; ^3^ Division of Biology, Department of Earth System Science, Faculty of Science, Fukuoka University, Fukuoka 814-0180, Japan

**Keywords:** antennae, single sensillum recording, entomopathogenic fungi, mutual grooming behavior

## Abstract

The termite *Coptotermes formosanus* Shiraki (Isoptera: Rhinotermitidae) protects itself from entomopathogenic fungus by mutual grooming behavior. *C. formosanus* removes foreign organisms, such as fungal conidia, from the body surface of its nestmates by mutual grooming behavior and eating them. The conidia removal rate from the body surface differed according to the isolate of entomopathogenic fungi (*Beauveria brongniartii* 782, *Paecilomyces fumosoroseus* K3, and *Metarhizium anisopliae* 455), and the removal rate of the fungal isolates seemed to depend on feeding preference, which was detrmined using paper discs moistened with a fungal suspension. In addition, it was found that *C. formosanus* without antennae groomed their nestmates more frequently than those with antennae. Consequently, it seems that *C. formosanus* antennae detect substances without touching, such as via odor, and it affects the efficiency of grooming behavior. The results of single sensillum recording support the hypothesis that *C. formosanus* are capable of distinguishing three species of fungi by their odors.

## Introduction

The termite *Coptotermes formosanus* Shiraki (Isoptera: Rhinotermitidae) is an important pest in Japan as it causes serious damage to wood and cellulose products (Vargo et al. 2003). With concerns over its environmental hazards and the possible development of pesticide-resistant termites, it is necessary to develop biological control strategies instead of chemicals to manage termite populations (Culliney and Grace 2000). Recently, research involved in the development of insect control with fungal agents has increased and 2.3% biopesticides used commercially are aimed at Isoptera (Faria and Wraight 2007). According to Yoshimura and Takahashi (1998), *Beauveria brogniartii*-infected workers of *C. formosanus* could kill approximately equal numbers of untreated individuals by contagion. On the other hand, there are a considerable number of reports about high termite resistance against fungal epizootics (Boucias et al. 1996; Rosengaus et al. 1998; Myles 2002; Shimizu and Yamaji 2003; Meikle et al. 2005). This disagreement is partly due to differences in quantity and quality aspects of the research, such as infection methods, conidia concentrations, or number of treated individuals. Behavioral interactions between termites and entomopathogenic fungi are still ambiguous, and it is useful to throw light on the relationship between termites and entomopathogenic fungi in an effort to develop sustainable biological pest control.

The insect cuticle is the first barrier that entomopathogenic fungi must overcome. Termite workers remove foreign organisms, such as fungal conidia, from the cuticle of their nestmates by grooming behavior, and fungal infection is prevented at the first stage of infection (Shimizu and Yamaji 2003; Yanagawa and Shimizu 2007). Myles (2002) stated that an infected individual was groomed more by their nestmates than healthy individuals. Since worker termites are blind, they sense their living environments by auditory and chemical means (Watson and Gay 1991). Antennal contact with nestmates is suggested to be essential for the induction of most social behaviors in termites (Dhanarajan 1980), and it was reported that antennae play a major role in the mediation of reaction to surrounding odors (Abushama 1964). Based on these reports, antennae of *C. formosanus* were studied here to clarify how they are used during grooming behavior.

Host perception of pathogens by olfactory cues is well studied in *Caenorhabditis elegans* (Zhang 2008), but little is known of this ability in insects. Further study is needed to fully understand the defense strategy of grooming behavior by termites against entomopathogenic fungi. The interaction between the fungal removal and its odor in grooming behavior was studied by Yanagawa et al. (2009). This study covers more expanded data than the previous paper and shows clearly that *C. formosanus* sense volatile substances emitted from fungal conidia on the cuticle of their nestmates before they touch each other and that the sensory apparatus on antennae are involved in the efficiency of grooming behavior. This paper provides the first single recording of impulses from antennal sensilla of *C. formosanus* responding to entomopathogenic fungal odors.

## Materials and Methods

### Insects

The termites, *C. formosanus,* used in this study were collected from one colony in Fukuoka, Japan and were maintained in plastic boxes (49 × 36 × 32 cm) in a dark chamber at 25° C; they were fed on seasoned pinewoods (kuromatsu; *Pinus thunbergii*). Workers were transferred from the above colonies into 90 × 15 mm Petri dishes containing a wet paper disc (No 2. Qualitative Filter Paper (90 mm dia.), Toyo Roshi Kaisha Ltd., www.advantec.co.jp/) and placed in a dark chamber at 25° C for one to three weeks before use.

### Preparation of conidial suspensions

Entomopathogenic fungi, *Beauveria* *brongniartii* 782, *Paecilomyces fumosoroseus* K3, and *Metarhizium anisopliae* 455 were maintained, and 1.0 × 10^7^ conidia/ml conidia suspensions were prepared as described in Yanagawa et al. (2009). FITC-labeled conidia suspensions were described as A-series suspensions, and unlabeled conidia suspensions were described as B-series suspensions.

### Detection of fungal conidia on cuticle of *C. formosanus*

In order to know the removal pattern of three fungal isolates, conidia attached to the cuticle after inoculation were detected in the same way as in the previous study (Yanagawa et al. 2008). Termites were submerged in an FITC-labeled conidial suspension (A-series) for inoculation and dried on filter paper. After inoculation, they were partitioned into groups of 10 in 90 × 15 mm Petri dishes containing a wet paper disc and incubated in a dark chamber at 25° C. At 0, 3, 6, and 24 h post-treatment, they were carefully mounted in a drop of Vectashield (Vector Laboratories, www.vectorlabs.com) to stabilize the fluorescence, and the conidia attached on their cuticle were counted with an epifluorescent microscope (Axioplan 2, Carl Zeiss, www.zeiss.com) at 200 x. The sum of five sites (head, thorax, and 2nd, 4th, and 6th abdominal segments) on each *C. formosanus* surface were calculated for the number in a square millimeter to estimate the amount on the whole body.

### Grooming behavior of *C. formosanus* with/without antennae

To clarify how antennae affect grooming behavior, this behavior was examined for termites with or without antennae that had been inoculated with fungal conidia. To prepare termites without antennae, both antennae were cut off at the scape after termites had been cold anesthetized on ice for 30 min. Termites with no antennae were reared in 90 × 15 mm Petri dishes containing a wet paper disc in a dark chamber at 25° C for over one week to allow the antennal cut end to heal. Termites with intact antennae were also anesthetized on ice for 30 min and reared for the same period as those without antennae in order to obtain control data. Thereafter, they were inoculated with *M. anisopliae* 455 conidial suspension (B-series) as described above. After inoculation, five termites were each put into Petri dishes (35 × 15 mm) and covered with a cardboard box during the experiment to reduce a room light effect on movements. Termites were left for 15 min to reduce the impact of treatment. Since it is impractical to observe and estimate the level of the grooming behavior for its whole duration, the frequency of touching each other was counted on photographs taken every 30 s for 30 min. Thus, total sixty photographs were taken per dish. Only the *C. formosanus* whose mouthparts touched their nestmates were counted. The number was estimated for each treatment with and without antennae inoculated with *M. anisopliae* 455 conidial suspensions or with a Tween 20 solution (control). Data obtained from 30 replicates and overall 600 termites were examined. The number of contacting termites was converted to the touching rate (%); total number of nestmate-touching termites/60 (all photographs).

### Feeding preference

Since the conidia removal due to grooming behavior in *C. formosanus* occurs by eating them (Yanagawa and Shimizu 2007), the preference for fungi was estimated by food intake rate (%) of the fungal-flavored filter paper disc, modifying the method of Ohmura et al. (2006). Though feeding rates could be affected by factors other than olfactory, as the discs used in this test had the same texture, the termites could not discriminate them by touch easily. Thus, especially at first, they should have discriminated between discs by utilizing antennal chemosensory receptors, which detected chemical cues emitted from conidial suspensions. To estimate the effects of the feeding activity on grooming behavior, the application time was decided as first 24 h in which the grooming behavior showed its benefit (Yanagawa and Shimizu 2005). Six quadrangular filter paper discs (7 × 7 mm) were placed on the bottom of Petri dishes (35 × 15 mm). The amount of food ingested was estimated by the following procedure. Three of six discs were treated with 150 µl test solution (B-series) containing 2 mg/ml of a blue food color (Blue Food Color No. 1, TCIFC: FO147, Tokyo Kasei Kogyo Co., LTD. www.tokyokasei.co.jp), and the others were treated with 150 µl colorless 0.025% Tween 20 solution (Treatment I). To avoid the influences of the food color material, the experiments were also conducted with opposite coloring (Treatment II) (Figure 2). Five workers were placed on the dish in each trial and allowed to feed on discs for 24 h. Thereafter, the *C. formosanus* in each dish were subjected to homogenization in 500 µl of 50% EtOH. After centrifuging twice at 15,000 rpm for 10 min, 150 µl of supernatant liquid was collected and the absorbance (Abs) of each was measured at 630 nm by an absorptiometer (DU-640E, Spectrophotometer, Beckman Coulter_TM_, www.beckmancoulter.com). The feeding preference of conidial suspension was calculated by the following equation: Feeding preference (Absorbance Index, AI) = (Abs Treatment I - Abs _Treatment II_) / Abs _Treatment II_. Data of 10 replicates, 100 *C. formosanus* per fungus, were pooled and analyzed per fungal isolate.

### Single sensillum recording

*C. formosanus* were fixed in a specimen holder made of a standard blue pipette tip (100–1000 µl) with wet cotton, and their antennae were arranged to stick out of the end of the tip, the orifice diameter of which had been fitted to the size of the termite. The cotton was moistened with physiological saline for cockroaches (200 m*M* KCl, 1.55%; 200 mM CaCl_2_-2H_2_O, 0.9%; 200 m*M* Na_2_HPO_4_-2H_2_O, 0.1%; 200 m*M* NaH_2_PO_4_-H_2_O, 0.9%) (Yamazaki and Narahashi 1959). An antennal platform was made of small piece of acrylic plate, and an antennal pillar was made of an electrolytically sharpened tungsten rod (0.5 mm in diameter) as protectors to prevent the antennae from moving during recording of electrical activities. The specimen was set on a research microscope (BX51WI, OLYMPUS) with the protectors. The active electrode, which was also an electrolytically sharpened tungsten rod, was inserted into the base of a sensillum, enough to make an electrical contact. The indifferent electrode, a silver wire (0.2 mm in diameter), was set in the specimen holder to contact the wet cotton. Electrical events were recorded using standard equipment.

The stimulus and control air were prepared as described in Yanagawa et al. (2009). Briefly, fresh air from the pure air source was divided to the two stream routes, a control stream and a stimulus stream, which were switched by two electric valves for stimulation, and the small glass bottles (30 ml) were set between the two routes. Each bottle contained a different kind of odor substance. One bottle contained 1 ml of 0.025% Tween 20 solution without any suspension. Each of the others contained 1 ml B-series conidial suspensions; *B. brongniartii* 782, *P. fumosoroseus* K3, or *M. anisopliae* 455. The air passing through these bottles was separately fed to glass tubes for stimulation. The specimen was exposed to the control clean air during the interstimulus time.

**Figure 1.**
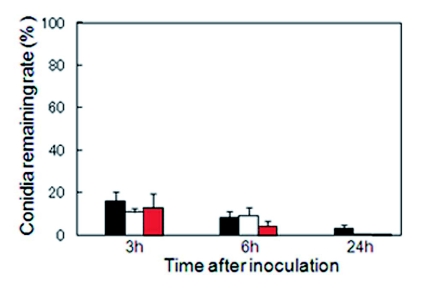
Persistence of conidia on the *Coptotermes formosanus* cuticle. (black): *Metarhizium anisopliae* 455 conidia remaining rate on the cuticle, (white): *Paecilomyces formosanus* K3 conidia remaining rate on the cuticle, (red): *Beauveria brongniartii* 782 conidia remaining rate on the cuticle. Y bar expresses the conidia remaining rate (%) on the cuticle; number of conidia on the cuticle at each time interval/number of conidia just after inoculation. Bars at top of columns represent standard errors. High quality figures are available online.

### Identification of sensillum

After impulse activities to a given odor were successfully recorded from sensilla, the regions of the sensilla were sketched in order to help locate the same sensilla on scanning electron microscope (SEM) observation. Samples were prepared by ultrasonic cleaning in 70% acetone, fixed in 4% (v/v) osminium acid for 2 h and dehydrated through an acetone series. They were then dried and coated with platinum-palladium using an ion spatter. Observations were carried out with a Hitachi S-4100 scanning electron microscope (www.hitachi-hta.com) equipped with a field emission gun.

### Statistical analysis

To examine the time-depended conidial reduction of each fungal species on the *C.* *formosanus* cuticle, analysis of variance (ANOVA) was applied to the mean number of conidia. The relationships expressed by linear regression (y: mean number of conidia attached to cuticle, x: time (h) postinoculation, r^2^: Peason's correlation coefficient) were obtained. Then, to compare the differences in conidia attachment and persistence on the *C. formosanus* surface among the three fungi, Poisson regression was applied to the counted data, and the KruskalWallis test was applied to assay the reduction rate. Grooming frequency data were analyzed by the Mann-Whitney test. Feeding preference was analyzed by Wilcoxon signed rank test to examine the fungal effects on *C. formosanus* feeding preference in each B-series conidial suspension . Then, multiple regression was conducted to compare the difference of those conidial suspensions by assuming AI = β_0_ + β_I_T_II_+ β_2_×1 + β3X_2_, where TII indicates the food color material; (X_1_, X_2_) is (0, 0) for *B. brongniartii* 782, (1, 0) for *M. anisopliae* 455, and (0, 1) for *P. fumosoroseus* K3. Thus β_2_ shows the effects of TII on AI, and β_2_ and β_3_ represents, respectively, the effect of *M.* *anisopliae* 455 and *P. fumosoroseus* K3 on AI when measured from *B. brongniartii* 782.

## Results

### **Detection of fungal conidia on cuticle of** *C.formosanus*

The binding of FITC-labeled conidia to the cuticle was quantified at 0, 3, 6, and 24 h after inoculation. The number of FITC-labeled conidia decreased markedly during the first 3 h after inoculation, and thereafter, the average number of conidia was less than 3/mm^2^ 24 h after inoculation in all genera of entomopathogenic fungi (Table 1). Attachment and persistence patterns among three genera of fungal conidia were statistically different (Poisson regression, p < .0001). Interestingly, the amount of conidia remaining on the cuticle was similar among three fungi when presented as a percentage of the amount just after inoculation (KruskalWallis test, p = 0.93). This suggests that the more conidia attached, the more disposed they are to remove them. However, the slope of linear regression was sharper for the conidia of *P. fumosoroseus* K3 and *B. brongniartii* 782 than for those of *M. anisopliae* 455 conidia: approximately 3.5% of *M. anisopliae* 455 conidia remained on the surface at 24 h post inoculation, but less than 1% of *P. fumosoroseus* K3 and *B. brongniartii* 782 (Figure 1).

**Figure 2.**
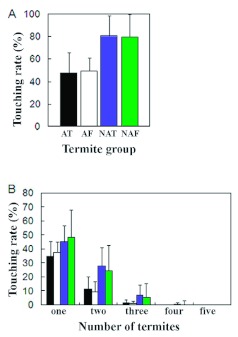
Grooming frequency of *Coptotermes formosanus* termites with/without antennae. A) Comparison of touching frequency of termite groups. AT (black): Termite group with intact antennae treated with Tween 20 solution. AF (white): Termite group with intact antennae treated with 1.0 × 10^7^/ml *M. anisopliae* 455 conidial suspension. NAT (blue): Termite group without antennae treated with Tween 20 solution. NAF (green): Termite group with intact antennae treated with 1.0 × 10^7^/ml *M. anisopliae* 455 conidial suspension. Bars on top of the columns represent standard errors. B) Details of touching condition in a dish. × bar expresses the number of termites, which touch a nestmate per dish. One means that one termite touches their nestmates in a dish. Y bar expresses the touching frequency (%); the *%* of touching termites in / all photographs. Bars on top of the columns represent standard errors. High quality figures are available online.

### Grooming behavior of *C. formosanus* with/without antennae

The touching frequency was similar between *C. formosanus* treated with Tween 20 solution and with conidia suspension of *M. anisopliae* 455 in the group of termites that had intact antennae (Mann-Whitney test, p = 0.212) and in the group that had no antennae (Mann-Whitney test, p = 0.796) (Figure 2A). The behavioral change was initiated by the presence of antennae, but not by the presence of entomopathogenic microbe. Termites without antennae touched their nestmates significantly more often than the group with antennae (Mann-Whitney test, p < .0001) (Figure 2A), and more termites in a dish participated in the mutual grooming behavior in the group without antennae (Figure 2B).

### Feeding preference

**Figure 3.**
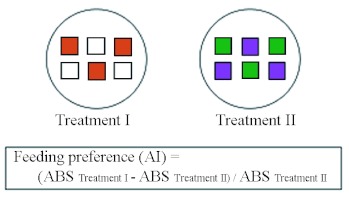
Feeding preference test. Treatment I,(orange): Paper disc containing 50 µl of 1.0 × 10^7^/ml conidia and 2 mg/ml blue food coloring suspended in Tween 20 solution, (white): Paper disc containing 50 µl of Tween 20 solution. Treatment II, (green): Paper disc containing 50 µl of 2 mg / ml blue food coloring suspended in a Tween 20 solution. (purple): Paper disc containing 50 µl of 1.0 × 10^7^/ ml conidia suspended in a Tween 20 solution. High quality figures are available online.

To estimate the feeding preference of *C. formosanus,* the ingested amounts of filter paper discs were compared between those given a disc containing a 0.025% Tween 20 solution only (control discs) and those given a disc containing B-series conidial suspensions of *M. anisopliae* 455, *P. fumosoroseus* K3, and *B. brongniartii* 782 (Figure 3).

*C. formosanus* consumed the *M. anisopliae* 455 disc significantly less than the control disc (Wilcoxon signed rank test: p = 0.002, Table 2a). In contrast, they consumed the *P. fumosoroseus* K3 disc significantly more than the control disc (Table 2a, Wilcoxon signed rank test: p = 0.055). Paper discs including *B. brongniartii* 782 were consumed at the same rate as control discs (Wilcoxon signed rank test: p = 0.688, Table 2a). The results of multiple regression analysis showed no influences of the food color material (p = 0.267 in TII parameter, Table 2b) and the isolate *P. fumosoroseus* K3 (p = 0.160 in X_2_ parameter, Table 2b). The significant difference appeared in the result of the isolate *M. anisopliae* 455 (p = 0.014 in X1 parameter, Table 2b). This means that termites stop eating significantly more when food material contained the conidia of *M. anisopliae* 455 compared with *B. brongniartii* 782 (estimate = -0.121 in X_1_ parameter, Table 2b). Moreover, in the previous study (Yanagawa et al. 2009), feeding behavior of *C. formosanus,* was enhanced when food material contained the conidia of *P. fumosoroseus* K3 compared with *B. brongniartii* 782, and the results in this study confirm this, although the change is not significant (estimate = 0.067 in X_2_ parameter, Table 2b). Therefore, *C. formosanus* rejected the fungal substances in the order of *M. anisopliae* 455 > *B. brongniartii* 782 > *P. fumosoroseus* K3. This tendency agrees with the conidia-remaining numbers and the rate on the surface at 24 h after inoculation (Table 1, Figure 2).

**Table 1.**
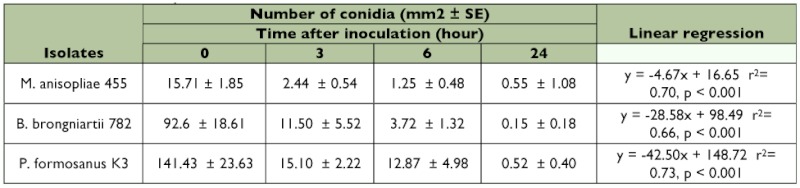
Attachment and persistence of conidia on termite surface

### Single sensillum recording

**Figure 4.**
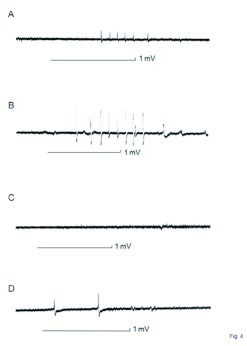
Recording examples of single sensillum responses of *Coptotermes formosanus* for odor stimuli originating from entomopathogenic fungi and Tween 20. A: Impulses responded to *Metarhizium anisopliae* 455, B: Impulses responded to *Paecilomyces fumosoroseus* K3, C: Impulses responded to *Beauveria brongniartii* 782, D: Impulses responded to Tween 20 (control). A horizontal bar with I mV vertical bar under each record is the stimulation mark. High quality figures are available.

Electrical activities were recorded from the sensilla responding to odors originating from these three kinds of fungi in order to examine whether *C. formosanus* recognize fungal odors and whether they distinguish the three genera of entomopathogenic fungi by antennal olfactory sensilla. *C. formosanus* antennal sensilla were randomly probed with the recording electrode to obtain stable recordings. Figure 4 shows examples of impulses responses to fungal odors recorded from the same single sensillum. The impulses seen in Figure 4A are small, and those in Figure 4B are large. The impulses in Figure 4D differ somewhat in shape from Figure 4A and 4B, indicating that at least three different receptor cells belong to the sensillum, each of which responds to one of the odors of *M. anisopliae* 455, *P. fumosoroseus* K3, and Tween 20 (control odor) but not to the odor of *B. brongniartii* 782. However, the sensilla from which responses were recorded did not necessarily show the same combination of receptor cells as described below.

The response profiles of the antennal sensilla to the odors of three entomopathogenic fungi and one control were investigated. Overall 327 sensilla from which responses to at least one of these four kinds of odors were successfully recorded are listed in Table 3. Whether one receptor cell responded to plural kinds of odors or whether receptor cells in a single sensillum each responded to different odors is not clear (see Table 3).

### Identification of sensillum

**Figure 5.**
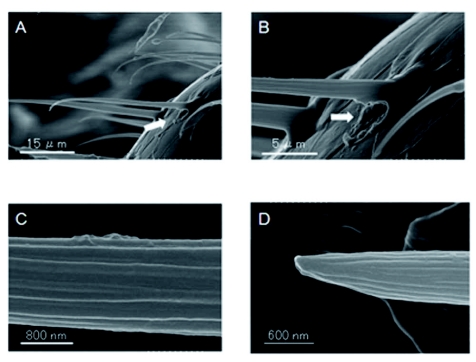
SEM picture of *Coptotermes formosanus* antennal hair responded to single sensillum recording. A: Identified sensillum chaetica. B: Basal part of the sensillum chaetica. C: Side of the sensillum chaetica. D: Tip of the sensillum chaetica. Arrow indicates the location of electrode tip insertion. High quality figures are available online.

About 50 sensilla from which responses had been successfully recorded were observed with scanning electron microscopy and identified (Figure 5). The hole at the base of the sensillum indicates the recording site in Figure 4B. All identified sensilla was chaetica in shape (Figure 5A). The sensilla had several longitudinal grooves on their cuticular surface, a single blunt tip, and a broad base (Figures 5B, 5C, 5D). The length of the cuticular apparatuses varied between 10 and 40 µm.

## Discussion

It is well known that termites protect themselves from fungal infection by means of mutual grooming behavior, however, the interactions between this behavior and fungal virulence are not clear. In this study, *C. formosanus* without antennae performed grooming behavior more frequently than those with intact antennae, and they removed conidia of those species of fungi to which they showed a higher feeding activity. Furthermore, impulse responses to entomopathogenic fungal odors were recorded from a single sensilla on *C. formosanus* antennae to examine whether they sense volatile substances emitted from fungal conidia and whether fungal odor is involved in the efficiency of grooming behavior.

Previously, it was reported that individually reared termites are susceptible to fungal infection in the order of *M. anisopliae* 455 > *B. brongniartii* 782 > *P. fumosoroseus* K3 (Yanagawa and Shimizu 2005). This order is roughly correlated with the order of the rate of conidia removal by intact *C. formosanus* (Figure 1). Fewer conidia of *M. anisopliae* 455 were removed from *C. formosanus* surface at 24 hours after inoculation than *B. brongniartii* 782 and *P. fumosoroseus* K3, irrespective of the number of attached conidia at inoculation (Figure 1 and Table 1). Furthermore, in the feeding preference test, discs containing the conidia suspension of *M. anisopliae* 455 were hardly consumed, but those of *P. fumosoroseus* K3 were consumed (Table 2). Consequently, it seems that fungal virulence has a strong correlation with the fungal removal by grooming behavior, and there is some interaction between grooming behavior and feeding activity.

**Table 2.**
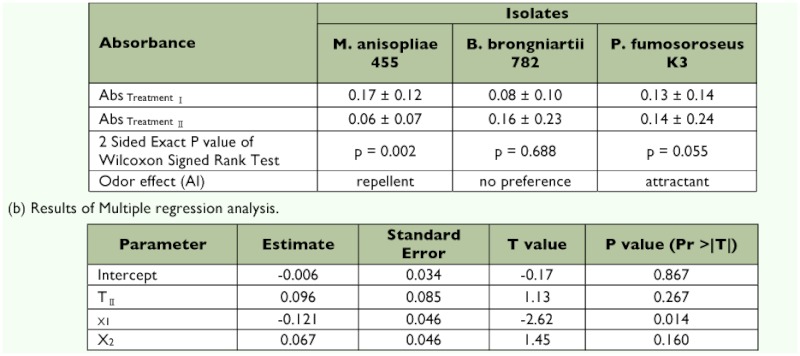
(a) Results of Feeding Preference Test.

The sensilla chaetica identified had several longitudinal grooves on their cuticular surface with a single blunt tip and a broad base (Figure 5). Although there are several studies on antennal sensilla of termites (Prestage et al 1963; Abushama 1964; Tarumingkeng et al. 1976; Costa-Leonardo and Soares 1997), it is difficult to compare the morphological features of the sensilla because different nomenclature was used. There are only a few physiological studies on the antennal sensilla of termites (Ziesmann 1996), and whether olfactory receptors or gustatory receptors were responsible for the discrimination of fungal conidia was not determined from the behavioral experiments here, however, the olfactory receptors in antennal trichoidal sensilla appeared to be largely involved in this discrimination in the electrophysiological study. Multiple combinations of fungal odors (Table 3) probably enable *C. formosanus* to distinguish among fungal conidia of different species by means of antennal olfactory information since fungal volatiles are species specific (Karlshøj et al. 2007). Termites without antennae died at a higher rate than those with antennae due to *M. anisopliae* infection in a preliminary experiment (Yanagawa et al. 2009) and touched each other more frequently than those with antennae (Figure 2). Considering these results, it seems that *C. formosanus* antennae affect the rate at which they associate with each other. Moreover, *C. formosanus* appear to have the sensory apparatus necessary to discriminate between fungal species, which in turn may explain the differential rates of conidia removal, which can influence the fungal virulence.

**Table 3.**
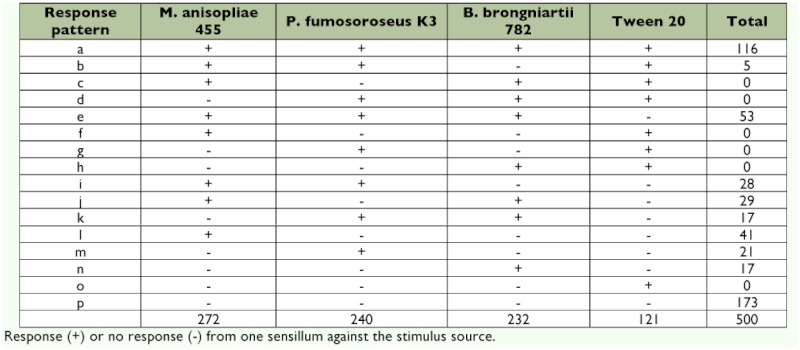
Response profile of termite single sensillum. Sensilla responsive patterns were classified according to the combination of stimulus source and by letters

Various virulence factors of fungi are involved in fungal infection of termites, and this virulence occurs by complicated processes and interactions (Clarkson and Charnley 1996). Important factors include: electrostatic and hydrophobic interaction between host cuticle and conidia, fungal germination polarity, fungi-secreted cuticledegrading enzyme, insect-host-like hydrocarbons, and effects of fungal second metabolites on the host insect (Leger et al. 1986; Boucias et al. 1988; Crespo and Cafferata 2000; Seger et al. 2005; Quesada-Moraga et al. 2006; Talaei-hassanloui et al. 2007). Nevertheless, as grooming behavior is very effective for conidia removal from the termite surface in the early stage of fungal infection, it largely contributes to disease resistance against fungal infection. Thus, it should be a focus for the development of biological controls to manage termite populations.

The reactions of termites to *B. brongniartii* 782 were unusual. In the feeding preference test, the suspension of this fungus was preferred by the termites to a similar extent as the control suspension (Table 2). On the other hand, it was most virulent to the termites among the three fungi in a high-density rearing condition, but not so virulent in a lowdensity rearing condition (Yanagawa and Shimizu 2005). Nevertheless, the conidia of *B. brongniartii* 782 on the cuticle were markedly removed, as shown in Table 1. The effective removal of *B. brongniartii* 782 conidia may be due to their attachment pattern, since many conidia of *B. brongniartii* 782 adhered directly to bristles (Yanagawa et al. 2008). Moreover, five sensilla did not respond to the odor of *B. brongniartii* 782 but responded to other stimuli (response pattern b, Table 3). The volatile substances emitted from *B. brongniartii* 782 might contain a component that inhibits the electrophysiological response of the antennae. These results indicate that disease resistance due to mutual grooming behavior does not work effectively for isolates that hardly induce a reaction. It is likely that *B. brongniartii* 782 develops a potent infection method, not in the attachment stage, but in another infection stage.
